# Heart Rate Variability Analysis on Electrocardiograms, Seismocardiograms and Gyrocardiograms on Healthy Volunteers

**DOI:** 10.3390/s20164522

**Published:** 2020-08-13

**Authors:** Szymon Sieciński, Paweł S. Kostka, Ewaryst J. Tkacz

**Affiliations:** Faculty of Biomedical Engineering, Department of Biosensors and Processing of Biomedical Signals, Silesian University of Technology, Roosevelta 40, 41-800 Zabrze, Poland; pkostka@polsl.pl (P.S.K.); etkacz@polsl.pl (E.J.T.)

**Keywords:** heart rate variability, electrocardiography, seismocardiography, gyrocardiography, accelerometers, gyroscopes

## Abstract

Physiological variation of the interval between consecutive heartbeats is known as the heart rate variability (HRV). HRV analysis is traditionally performed on electrocardiograms (ECG signals) and has become a useful tool in the diagnosis of different clinical and functional conditions. The progress in the sensor technique encouraged the development of alternative methods of analyzing cardiac activity: Seismocardiography and gyrocardiography. In our study we performed HRV analysis on ECG, seismocardiograms (SCG signals) and gyrocardiograms (GCG signals) using the PhysioNet Cardiovascular Toolbox. The heartbeats in ECG were detected using the Pan–Tompkins algorithm and the heartbeats in SCG and GCG signals were detected as peaks within 100 ms from the occurrence of the ECG R waves. The results of time domain, frequency domain and nonlinear HRV analysis on ECG, SCG and GCG signals are similar and this phenomenon is confirmed by very strong linear correlation of HRV indices. The differences between HRV indices obtained on ECG and SCG and on ECG and GCG were statistically insignificant and encourage using SCG or GCG for HRV estimation. Our results of HRV analysis confirm stronger correlation of HRV indices computed on ECG and GCG signals than on ECG and SCG signals because of greater tolerance to inter-subject variability and disturbances.

## 1. Introduction

Physiological variation of the interval between consecutive heart beats caused by the activity of the autonomic nervous system is known as heart rate variability (HRV). The analysis of heart rate variability found its use in the diagnosis of different clinical and functional conditions in the last decade [1,2]. HRV analysis is traditionally performed on inter-beat intervals obtained from the R waves in electrocardiograms [3,4]. The development of high quality, sensitive and inexpensive sensors based on microelectromechanical systems (MEMS) helped rediscover the clinical potential of the analysis of cardiac vibrations on the chest wall [5,6]. The analysis of cardiac vibrations consists of two complementary methods: Seismocardiography (SCG) and gyrocardiography (GCG) [7,8].

Seismocardiography is a non-invasive technique of recording and analyzing cardiac activity by measuring precordial acceleration [9]. The registered signal called seismocardiogram is a non-stationary signal with morphological variations between subjects [5]. In the past, SCG was mainly used by physiologists due to the need of complex recording devices [10]. Technological improvements and miniaturization of accelerometers make seismocardiography a useful non-invasive technique for examining cardiac activity [5,11,12,13,14]. The applications of SCG include heart monitoring during magnetic resonance imaging (MRI) scan [15,16], monitoring response to cardiac interventions [5] heart rate variability analysis [4,13,17,18,19,20], the detection of atrial fibrillation [6,21,22], heart failure [22,23] and the diagnosis of myocardial ischemia [5,24,25,26].

Gyrocardiography is a non-invasive technique for acquisition and analysis of changes in angular velocity of the chest associated with cardiac activity. The gyrocardiography was introduced by Tadi et al. [7,8,27]. The GCG found its use in the analysis of mechanical activity of the heart [8], including the cardiac function of dogs [28], heart rate (HR) [29,30,31] and pre-ejection period estimation [21] and also the diagnosis of cardiovascular disesases [22,23,32], including atrial fibrillation [21,22,33], coronary artery disease [34] and acute decompensated heart failure [22]. In the last years gyrocardiography and seismocardiography are becoming a viable alternative to electrocardiography (ECG) due to the cost effectiveness and increasing accuracy [31].

The first HRV analysis on ECG signals and cardiac mechanical signals (mechanocardiograms) was performed by Friedrich et al. in 2010 [35] on ballistocardiograms. In 2012 Ramos-Castro et al. performed the first HRV analysis on seismocardiograms [13] and Laurin et al. [3] proved the validity of HRV indices obtained from SCG signal in 2013. Since then, the HRV analysis on seismocardiograms was also performed by Tadi et al. [4], Landreani et al. [17,18], Siecinski et al. [19,20,36,37,38]. The study described by Lahdenoja et al. [39] was the first attempt to perform HRV analysis using GCG signals. Gyrocardiograms (GCG signals) were used to improve the reliability of HRV analysis on mechanocardiograms. Iftikhar et al. [32] and Mehrang et al. [22] crafted features based on heart rate variability on mechanocardiograms to classify between healthy subjects and patients suffering from atrial fibrillation, coronary artery disease and acute decompensated heart failure.

In this study we compare the results of heart rate variability analysis performed on electrocardiograms, seismocaridograms and gyrocardiograms acquired from healthy volunteers. The results of HRV analysis are presented as time domain and frequency domain HRV indices and the indices which describe Poincaré maps. The differences between the HRV indices calculated on ECG, SCG and GCG signals were expressed as absolute errors and Pearson’s linear correlation coefficients. In this way we attempt to cross-validate the beat-to-beat variations in SCG and GCG with ECG as a reference.

## 2. Materials and Methods

### 2.1. Data Set

We performed the analyses on “Mechanocardiograms with ECG Reference” data set by Kaisti et al. [40] publicly available at IEEE DataPort data repository [41]. The data set consists of 29 simultaneous recordings of ECG, SCG and GCG signals registered on 29 healthy male volunteers with the following demographics (minimum, maximum, mean, standard deviation): age (23–41, 29, 5 years), height (170–190, 179, 5 cm), weight (60–98, 76, 11 kg) and BMI (18–30, 24, 3.00 kg/m^2^) [40]. The data were acquired between the 4 April 2014 and 2 November 2015 [41] and the total length of recoded signals is about 260 min [40]. In Table 1 we provide a short description of each recording based on the annotations in the data set [41].

The data were recorded simultaneously with the sampling frequency of 800 Hz and sensors were attached to the sternum with the double-sided tape. During the study the volunteers were lying either in the supine position or on their left or right side [39,40,41]. The axes of rotations/translations were defined as follows: The *x*-axis was oriented laterally from left to right, the *y*-axis points was oriented from head to foot and the *z*-axis was oriented from back to chest.

Seismocardiograms were obtained by a triple-axis capacitive digital accelerometer (MMA8451Q from Freescale Semiconductor, Austin, TX, USA), gyrocardiograms were acquired by MAX21000 gyroscope (Maxim Integrated, San Jose, CA, USA) 3-axial gyroscope and electrocardiograms were obtained for reference by ADS1293 from Texas Instruments [8,40]. As stated in [40], the study on acquisition of ECG, SCG and GCG signals was conducted in accordance with the Declaration of Helsinki and the study design was approved by the Ethical Committee of the Hospital District of Southwest Finland.

### 2.2. Signal Processing

Signal processing and algorithm development was performed in Matlab R2018b. The recordings saved as text files [41] were imported into Matlab. Due to the spikes and baseline wandering the ECG signals in recordings 15–20, 23, 25, 27 and 29 were preprocessed as follows:

The first step was to remove the spike at the first 3–17 samples of the signal. The next step was applying the 5th order median filter. To remove the baseline wander in recordings 20, 21, 27 and 29 we applied Empirical Mode Decomposition (EMD) due to its effectiveness [42].

The implementation of the EMD algorithm in MATLAB R2018b applies the maximum number of iterations and the sifting tolerance as stop criteria of sifting process. The stop criterion of the empirical mode decomposition is the signal to residual energy ratio [43]. The maximum number of IMFs was set to 10, the number of sifting iterations was limited to 100 and the piece-wise Hermite interpolating polynomial was used to perform interpolation. In recording 20 the baseline wandering of the electrocardiogram was removed by the reconstructing the original signal without the residual. Baseline wander was removed in recording 21 by removing the first 9566 samples of the signal and reconstruction of the signal by the addition of IMFs numbers 2–9.

Then, the R waves in ECG signals were detected with Pan–Tompkins algorithm [44] implemented in MATLAB by D. Wedekind [45].

#### 2.2.1. Heartbeat Detection in ECG

Heartbeat detection in ECG signals is based on nearly periodic occurrence of R waves [46]. To detect ECG R waves in ECG we applied Pan–Tompkins algorithm which consists of the following steps: Bandpass filtering, differentiation, squaring of samples, smoothing with a moving average filter, correlation analysis and thresholding [44]. The intervals between consecutive heartbeats (inter-beat intervals) are calculated as follows:

(1) $$t_{RR,i}=t_{n}-t_{n-1}$$

where $t_{RR,i}$ is the *i*-th cardiac interval in ECG and $t_{n}$ denotes the occurrence of *n*-th R wave.

#### 2.2.2. Heartbeat Detection in SCG and GCG

Heartbeat detection is based on the fact of quasi-periodic occurrence of waves associated with heartbeats [8]. The wave which indicates the heartbeat in seismocardiograms and gyrocardiograms is the aortic valve opening (AO) wave [28,30] also known as the $g_{J}$ wave in GCG signal morphology [8]. The AO wave occurs within 100 ms after the occurrence R wave in ECG signal [8,47]. Due to the high signal-to-noise (SNR) ratio, we analyzed only the *z*-axis of the SCG signal [24,48] and *y*-axis of the GCG signal [7,8,29,33,40].

SCG and GCG signals did not require preprocessing before beat detection because band pass filtering was sufficient to remove the baseline wander and the offset due to the influence of the gravitational acceleration. In order to detect heartbeats, we applied the algorithm described in [4] which uses ECG R waves as reference points for detection of heartbeats in seismocardiograms and gyrocardiograms and is based on the windowing method proposed in [15,49]. We used this approach because of the inter-subject variation in the SCG signal morphology and its susceptibility to motion artifacts [4,24] and to verify its feasibility in case of GCG signals.

The first step is bandpass filtration with the third-order Butterworth bandpass filter with a passband of 4–50 Hz. Then, SCG and GCG signals are smoothed using a moving average filter with the window width of 15 ms. The moving average filter was implemented as a zero-phase finite impluse response (FIR) filter. Local maxima within 100 ms since the occurrence of ECG R waves are considered as occurrences of the AO/$g_{J}$ waves.

The *i*-th inter-beat intervals in SCG and GCG signals is calculated as:

(2) $$t_{AO-AO,i}=t_{n}-t_{n-1}$$

where $t_{n}$ denotes the occurrence of *n*-th AO wave in SCG and GCG signal.

**Figure 1:**
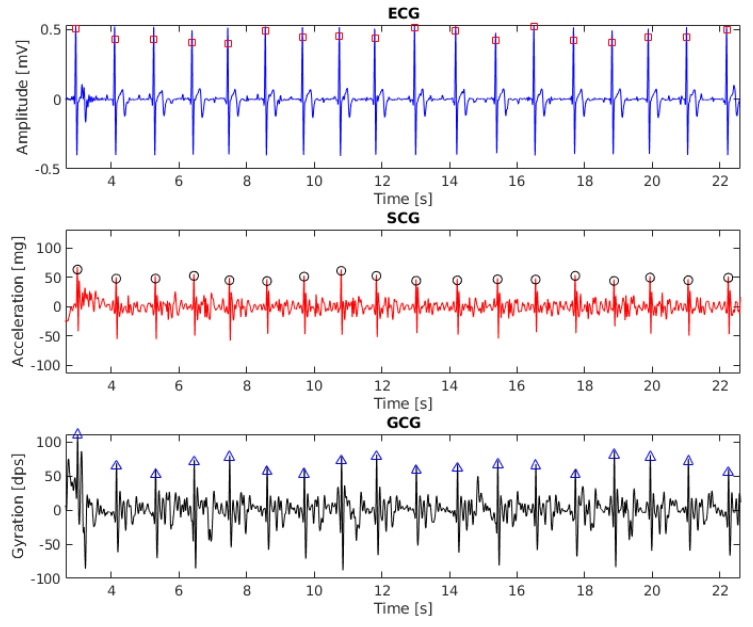
Electrocardiogram (ECG), seismocardiogram (SCG) and gyrocardiogram (GCG) signals with annotated heartbeats (subject 6, 20 s fragment). Detected heartbeats are shown as squares (for ECG), circles (for SCG) and triangles (for GCG).

The results of beat detection in ECG, SCG ang GCG signal (recording 6, 20 s fragment) are shown in Figure 1. To align the baseline with zero the signals were filtered with the third-order Butterworth bandpass filter with cut-off frequencies of 1 Hz and 40 Hz.

### 2.3. HRV Analysis

The HRV analysis was performed in time domain and frequency domain with PhysioNet Cardiovascular Toolbox which was developed as a part of the study by Vest et al. on open-source HRV analysis tool [50] and is licensed under the GNU General Public License 3 [51].

The simplest method of HRV analysis is time domain analysis which is applied to the series of successive normal inter-beat intervals [52]. The normal inter-beat interval (NN) is defined as the interval between consecutive R waves in ECG signals, AO waves in SCG signals or AO/$g_{J}$ waves in GCG signals. We considered mean inter-beat intervals (AVNN), the standard deviation of the NN intervals (SDNN), the root mean square of successive differences of NN intervals (RMSSD) and the proportion of the number of adjacent NN intervals whose durations differ more than 50 ms with respect to the total number of NN intervals (pNN50). SDNN depends on the length of the signal [52] and its clinical significance is the measure of cardiac risk [53]. RMSSD is related clinically to respiratory sinus arrhythmia (RSA) and high frequency changes in the heart rhythm in response to respiration [53,54]. The pNN50 index is correlated with the activity of parasympathetic nervous system [54].

Frequency domain HRV analysis was based on calculating the power of the HRV frequency band as the 1024-sample Lomb periodogram [50,51]. We used the following indices: VLF which is the power of the very low frequency band (0.0033–0.04 Hz), LF which is the power of the low frequency band (0.04–0.15 Hz) and the power of the high frequency band (HF) computed in the band 0.15–0.4 Hz. The LF/HF ratio was obtained as the LF/HF ratio and represents the balance of autonomic nervous system [50,52]. Total power (TP) is calculated as the power of the frequencies up to 0.4 Hz and reflects the variance of all NN intervals [52]. The power of the low frequency (LF) band represents the activity of the sympathetic nervous system whereas the power of the high frequency band is related to the parasympathetic nervous system [52,54].

Despite conducting the research on heart rate variability since 1965 [55] there is no consensus on how to calculate HRV coefficients in scientific research and clinical practice. That makes the comparison of results of the HRV analysis found in various publications difficult [56,57]. One of the proposed solutions is to use software accepted in clinical practice, such as Kubios [56] or to develop free and open source software for comprehensive HRV analysis which uses raw signals related to the heart activity or inter-beat intervals [57]. That is the reason of using PhysioNet Cardiovascular Signal Toolbox for our analyses.

The HRV analysis was performed on full inter-beat interval signals and the inter-beat interval signals divided into 179 s overlapping time windows with a 15 s step. We define full inter-beat interval signals as the signals of inter-beat interval signals derived from full length ECG, SCG or GCG signals. We performed the HRV analysis in two variants (full inter-beat interval signals and inter-beat interval signals divided into overlapping time windows) to assess the influence of dividing signals into overlapping windows on the HRV indices. The length of time window was 179 s because the shortest recordings have the length of 3 min. The preprocessing of inter-beat intervals divided into overlapping time windows is further described in [50].

#### Poincaré Maps

**Figure 2:**
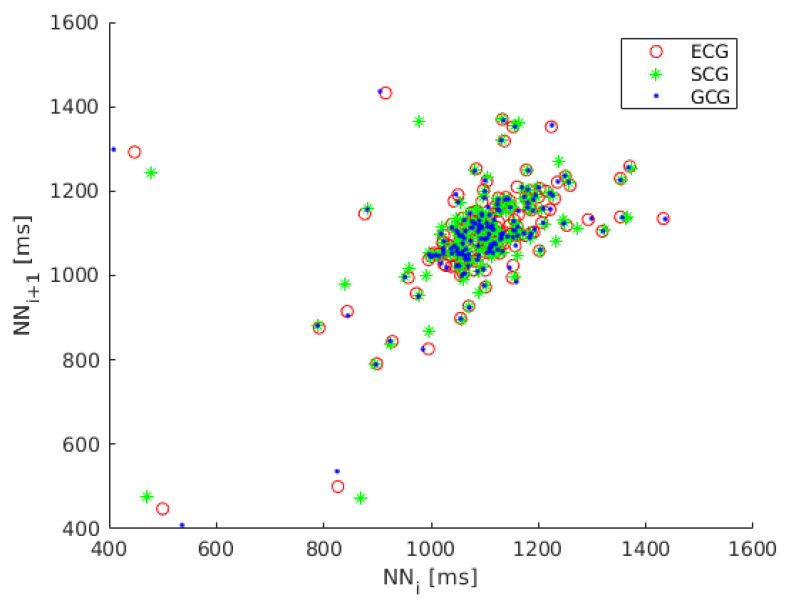
Poincaré map of heart rate variability (HRV) calculated on ECG (red circles), SCG (green asterisks) and GCG signal (blue dots).

Because the dynamics of heart rate variability is nonlinear we also considered the analysis of Poincaré maps, a geometrical technique taken from nonlinear dynamics [58,59,60]. The Poincaré map (Lorenz plot) is the scatter plot of inter-beat intervals as a function of previous inter-beat intervals [61]. The Poincaré map plotted for recording 1 is shown in Figure 2.

The Poincaré map may be described by several indices, such as $SD_{1}$, $SD_{2}$, $SD_{1}/SD_{2}$, VAI (vector angular index) and VLI (vector length index). Fitting an ellipse to the shape of Poincaré plot and measuring the dispersion of scatter points is a popular approach in analysis of Poincaré maps [60,62].

$SD_{1}$ reflects the short term variability of heartbeats and is defined as the width of the ellipse calculated as the standard deviation of the distances from the identity line (y=x axis):

(3) $$SD_{1}=stddev\left(\frac{\left|NN_{i+1}-NN_{i}\right|}{\sqrt{2}}\right)$$

where $NN_{i}$ is an *i*-th inter-beat interval series for i=1,2,⋯N−1, $NN_{i+1}$ is the next inter-beat interval.

$SD_{2}$ defined as the length of the ellipse measures the long term variability of heartbeats and is calculated as the standard deviation of the distances of points from $y=-x+2\bar{NN}$ axis:

(4) $$SD_{2}=stddev\left(\left|\frac{NN_{i+1}-NN_{i}}{\sqrt{2}}-2\bar{NN}\right|\right)$$

where $NN_{i}$ and $NN_{i+1}$ are defined as in Equation (3), $\bar{NN}$ is the mean of all inter-beat intervals and stddev() denotes the standard deviation [58,62].

$SD_{1}/SD_{2}$ is the measure of the randomness in HRV time series and is defined as the ratio of $SD_{1}$ to $SD_{2}$ [63]. Vector Angular Index (VAI) and Vector Length Index (VLI) are the measures of dispersion of scatter points in Poincaré maps proposed by Ruan et al. to detect atrial fibrillation based on the irregularities of inter-beat intervals [62].

VAI measures the angular dispersion of scatter points and the VLI measures the distance dispersion of scatter points. VAI is calculated as the mean of all the absolute value of angular differences between the lines plotted from every scatter point to the original point (0,0) and the identity line:

(5) $$VAI=\frac{1}{N}\sum_{i=1}^{N}\left|\theta_{i}-45^{\circ }\right|$$

where $\theta_{i}$ is the angle between the line plotted from *i*-th scatter point to the original point in degrees and the *x*-axis and *N* is the number of scatter points.

VLI is specified as the standard deviation of all distances of scatter points from the original point:

(6) $$VLI=\sqrt{\frac{1}{N}\sum_{i=1}^{N}{\left(l_{i}-L\right)}^{2}}$$

where $l_{i}$ is the length between *i*-th scatter point and the original point and *L* is the mean of all $l_{i}$ [20,62].

## 3. Results

The results of HRV analysis in time domain, frequency domain and based on Poincaré maps on ECG, SCG and GCG signals expressed as mean and standard deviation (SD) values of HRV indices are shown in Table 2 on entire signals and in Table 3 on signals divided into overlapping time windows. Mean and standard deviation values of HRV indices obtained on electrocardiograms, seismocardiograms and gyrocardiograms are very similar in both variants (full signals and signals divided into overlapping time windows).

The absolute error of HRV indices obtained on SCG and GCG signals was calculated to express the similarity to the reference values calculated on electrocardiograms. Table 4 presents the mean and standard deviation values of the absolute error of the HRV indices obtained on full signals. Table 5 shows the mean and standard deviation values error of the absolute error of HRV coefficients for inter-beat intervals divided into overlapping 179 s time windows.

Standard deviations of relative errors in Table 4 and Table 5 are larger than the mean values which indicates large discrepancies. Relative errors between HRV indices calculated on ECG and GCG signals are smaller than for HRV indices calculated on ECG and SCG signals in both cases (full HRV signals and HRV signals divided into overlapping 179 s time windows). The smallest relative error is observed for AVNN and the largest relative error is observed for pNN50.

According to observations in Tadi et al. study [4], HRV indices obtained from SCG and ECG signals have strong linear relationship. Because seismocardiography and gyrocardiography are complementary techniques [7], the correlation between the results of HRV analysis performed on ECG, SCG and GCG signals should be strong. The strength of linear correlation between each analyzed HRV index calculated on ECG, SCG and GCG signals was expressed as Pearson correlation coefficient (ρ). The results presented in Table 6 and Table 7 indicate very strong linear correlation for the *p*-value under 0.001. We define a strong linear correlation between two analyzed data sets for R > 0.7 [64].

## 4. Discussion

We found insignificant differences between the HRV indices calculated on electrocardiograms, seismocardiograms and gyrocardiograms calculated on signals divided into overlapping windows and full length of signals acquired on healthy subjects. That fact confirms the possibility of conducting HRV analysis on seismocardiograms [3,4,13,19,20,36] and gyrocardiograms [39] as well as on electrocardiograms. C. Yang et al. [23], Z. Iftikhar et al. [32] and S. Mehrang et al. [22] use heartbeat detection and heart rate variability to craft features in classification of cardiovascular diseases. However, they did not concentrate on HRV analysis and that fact hampers the comparison of the results of HRV analysis on healthy patients and patients suffering from cardiovascular diseases.

HRV indices obtained on electrocardiograms were treated as a reference for HRV coefficients calculated on seismocardiograms and gyrocardiograms as suggested by the work of m, Laurin et al. [3], Tadi et al. [4], Lahdenoja et al. [39] and Sieciński et al. [19]. Mean and standard deviation values of HRV coefficients calculated on full HRV signals are similar to the values reported on young people by Shlyk et al. [65], Keet et al. [66] and Corrales et al. [67] for all analyzed signals (ECG, SCG, GCG). The values of HRV coefficients obtained on full signals are significantly higher than the values calculated on signals divided into overlapping windows except AVNN, pNN50, LF/HF, *SD*_1_/*SD*_2_. The reason is that these indices are independent from the length of the analyzed signal.

The HRV coefficients calculated in our study on the seismocardiograms presented in Table 2 are slightly diffrent than reported by Charlier et al. [68], Ramos-Castro et al. [13], Sieciński et al. [19,36]. Mean differences between the values of analyzed HRV coefficients calculated on the ECG, SCG and GCG signals are small in comparison with the mean values. Mean and standard deviation of relative errors of HRV indices are smaller for GCG signals than for SCG signals. Pearson linear correlation coefficients indicate a stronger compatibility of HRV coefficients calculated on gyrocardiograms and electrocardiograms than on seismocardiograms and electrocardiograms. These findings are in line with the observations of P.-F. Migeotte et al. [69] and C. Yang and N. Tavassolian [23] on the accuracy of classification of cardiovascular abnormalities on SCG and GCG signals. Based on their observations, the GCG signal is more tolerant to inter-subject variability [8] and disturbances than the SCG signal [7,70] because over 60% of the total kinetic energy transfered from the heart to the chest wall is the rotational energy [69].

HRV indices calculated on gyrocardiograms are similar to those obtained on electrocardiograms and seismocardiograms because of the synchronization of electrical activity of the heart registered in ECG and mechanical cardiac activity reflected in seismocardiograms and gyrocardiograms [8,47,71]. While there is no direct relationship between the occurence of the AO/$g_{J}$ wave in SCG and GCG signals and the R wave in the electrocardiograms, the 100 ms delay between them is well described in [8,47,49].

Correlation coefficients of HRV indexes calculated on electrocardiograms and seismocardiograms are higher than reported by Ramos-Castro et al. [13], Sieciński et al. [19,36] and lower than in the work of Tadi et al. [4] for all HRV indices except AVNN. Pearson’s linear correlation coefficient (ρ) was closer to 1.000 for HRV indices obtained on full signals than for HRV indices calculated on signals divided into overlapping windows.

The limitations of our study are the study group limited to healthy male subjects between 23 and 41 years of age and the dependence of heartbeat detection in SCG and GCG signals on the R waves in ECG. In future works we will consider the influence of different cardiovascular conditions and various independent heartbeat detectors in SCG and GCG signals and their accuracy on the agreement of HRV indices calculated on SCG and GCG signals with the gold standard (HRV indices obtained on electrocardiograms).

## 5. Conclusions

This study presents the feasibility of performing HRV analysis on electrocardiograms, seismocardiograms and gyrocardiograms. The findings support the idea that seismocardiography or gyrocardiography may be used to monitor heart rate and provide reliable HRV analysis. We also proved that we can detect heartbeats on gyrocardiograms by detecting the local maxima within 100 ms of the occurrence of an R wave in electrocardiogram. However, the results presented here were obtained using the ECG R waves as references for the identification of heartbeats in SCG and GCG. In practical situations, independent and reliable beat detectors are necessary for SCG and GCG.

## Figures and Tables

**Table 1:** Recording description.

Subject Number	Length of Recording	Remarks
1	3 min	Breathing: 2 min normal, 30 s holding a breath, 30 s normal
2	3 min	Breathing: 2 min normal, 30 s holding a breath, 30 s normal
3	3 min	Breathing: 2 min normal, 30 s holding a breath, 30 s normal
4	3 min	Breathing: 2 min normal, 30 s holding a breath, 30 s normal
5	3 min	Breathing: 2 min normal, 30 s holding a breath, 30 s normal
6	3 min	Breathing: 2 min normal, 30 s holding a breath, 30 s normal.
		Sensor not strictly secured on chest because of body hair.
7	3 min	Breathing: 2 min normal, 30 s holding a breath, 30 s normal
8	3 min	In supine position
9	10 min	In supine position
10	10 min	In supine position
11	30 min	In supine position
12	10 min	In supine position
13	10 min	In supine position
14	10 min	In supine position
15	10 min	In supine position
16	10 min	In supine position
17	10 min	In supine position
18	10 min	In supine position
19	10 min	In supine position
20	10 min	In supine position
21	10 min	In supine position
22	10 min	In supine position; sensor loose in the end.
23	10 min	
24	10 min	In supine position
25	9 min	In supine position
26	10 min	In supine position
27	10 min	
28	10 min	In supine position
29	10 min	In supine position

**Table 2:** Mean and standard deviation values of HRV indices calculated on full signals.

HRV Index	ECG	SCG	GCG
	Mean (SD)	Mean (SD)	Mean (SD)
AVNN [ms]	954.90 (113.36)	954.88 (113.36)	954.91 (113.37)
SDNN [ms]	84.18 (33.41)	86.7100 (31.60)	86.96 (33.42)
RMSSD [ms]	75.84 (41.16)	83.68 (36.37)	77.7177 (36.37)
pNN50	0.30 (0.19)	0.37 (0.17)	0.31 (0.19)
VLF [ms^2^]	1860.90 (1369.11)	1861.44 (1371.98)	1864.24 (1371.98)
LF [ms^2^]	2570.18 (2251.61)	2609.14 (2245.32)	2601.45 (2293.06)
HF [ms^2^]	2774.35 (2378.19)	2909.42 (2259.84)	2867.19 (2398.12)
LF/HF	1.2659 (0.8454)	1.0993 (0.7743)	1.2048 (0.8065)
TP [ms^2^]	8042.73 (5466.70)	8216.96 (5340.56)	8170.00 (5545.13)
*SD*_1_ [ms]	53.70 (29.16)	59.25 (25.7694)	55.0292 (28.95)
*SD*_2_ [ms]	105.35 (39.73)	106.66 (38.58)	105.9665 (39.79)
*SD*_1_/*SD*_2_	0.49 (0.15)	0.55 (0.14)	0.50 (0.15)
EA [ms^2^]	20,738.45 (16,359.85)	22,364.41 (15,599.28)	21,252.91 (16,489.52)
VAI [${}^{\circ }$]	1.39 (0.64)	1.61 (0.57)	1.4442 (0.65)
VLI [ms]	104.85 (39.71)	106.04 (38.73)	105.48 (39.79)

**Table 3:** Mean and standard deviation values of HRV indices calculated on divided into overlapping 179 s time windows.

HRV Index	ECG	SCG	GCG
	Mean (SD)	Mean (SD)	Mean (SD)
AVNN [ms]	941.23 (110.08)	941.0472 (110.00)	941.3313 (110.08)
SDNN [ms]	64.94 (22.09)	67.4271 (21.28)	65.6358 (22.32)
RMSSD [ms]	52.55 (23.89)	60.01 (22.51)	54.09 (23.25)
pNN50	0.27 (0.19)	0.34 (0.17)	0.28 (0.18)
VLF [ms^2^]	1645.09 (1505.34)	1646.89 (1514.59)	1663.31 (1554.52)
LF [ms^2^]	1575.98 (1131.11)	1617.99 (1162.03)	1608.56 (1176.83)
HF [ms^2^]	1343.52 (1024.29)	1493.9831 (1015.87)	1386.01 (1024.23)
LF/HF	1.70 (1.38)	1.41 (1.11)	1.57 (1.18)
TP [ms^2^]	4845.82 (3090.72)	5039.4577 (3093.67)	4941.80 (3195.36)
*SD*_1_ [ms]	37.26 (16.95)	42.55 (15.97)	38.35 (16.50)
*SD*_2_ [ms]	83.30 (28.22)	84.63 (27.77)	83.9605 (28.64)
*SD*_1_/*SD*_2_	0.44 (0.14)	0.5101 (0.1534)	0.46 (0.13)
EA [ms^2^]	10,835.40 (7491.69)	12,246.36 (7433.61)	11,192.33 (7649.02)
VAI [${}^{\circ }$]	1.18 (0.49)	1.38 (0.48)	1.2156 (0.47)
VLI [ms]	82.97 (28.03)	84.28 (27.59)	83.61 (28.45)

**Table 4:** Mean and standard deviation of relative error of HRV indices calculated on full signals.

HRV Index	ECG-SCG	ECG-GCG
	Mean (SD)	Mean (SD)
AVNN [ms]	0.00 (0.00)	0.00 (0.00)
SDNN [ms]	0.05 (0.09)	0.01 (0.01)
RMSSD [ms]	0.22 (0.36)	0.06 (0.13)
pNN50	2.87 (12.38)	0.88 (4.26)
VLF [ms^2^]	0.01 (0.01)	0.00 (0.00)
LF [ms^2^]	0.05 (0.07)	0.02 (0.02)
HF [ms^2^]	0.24 (0.45)	0.05 (0.06)
LF/HF	0.12 (0.16)	0.07 (0.09)
TP [ms^2^]	0.06 (0.12)	0.02 (0.01)
*SD*_1_ [ms]	0.22 (0.36)	0.06 (0.13)
*SD*_2_ [ms]	0.02 (0.04)	0.01 (0.01)
*SD*_1_/*SD*_2_	0.19 (0.29)	0.05 (0.12)
EA [ms^2^]	0.26 (0.44)	0.06 (0.13)
VAI [${}^{\circ }$]	0.25 (0.38)	0.06 (0.14)
VLI [ms]	0.02 (0.04)	0.01 (0.01)

**Table 5:** Mean and standard deviation of relative error of HRV indices calculated on signals divided into overlapping 179 s time windows.

HRV Index	ECG-SCG	ECG-GCG
	Mean (SD)	Mean (SD)
AVNN [ms]	0.00 (0.00)	0.00 (0.00)
SDNN [ms]	0.06 (0.09)	0.02 (0.02)
RMSSD [ms]	0.24 (0.40)	0.08 (0.23)
pNN50	0.81 (2.90)	0.15 (0.69)
VLF [ms^2^]	0.03 (0.05)	0.01 (0.03)
LF [ms^2^]	0.08 (0.08)	0.03 (0.04)
HF [ms^2^]	0.28 (0.53)	0.11 (0.25)
LF/HF	0.16 (0.16)	0.08 (0.10)
TP [ms^2^]	0.08 (0.12)	0.03 (0.03)
*SD*_1_ [ms]	0.24 (0.40)	0.08 (0.23)
*SD*_2_ [ms]	0.03 (0.04)	0.01 (0.01)
*SD*_1_/*SD*_2_	0.20 (0.33)	0.08 (0.22)
EA [ms^2^]	0.28 (0.48)	0.09 (0.24)
VAI [${}^{\circ }$]	0.24 (0.38)	0.07 (0.18)
VLI [ms]	0.03 (0.04)	0.01 (0.01)

**Table 6:** Pearson’s linear correlation coefficient of HRV indices obtained on ECG and SCG signals.

HRV Index	ρ (Full Signals)	ρ (Signals Divided into 179 s Windows)
AVNN	1.000	1.000
SDNN	0.993	0.979
RMSSD	0.965	0.886
pNN50	0.852	0.858
VLF	1.000	0.998
LF	0.997	0.973
HF	0.994	0.970
LF/HF	0.947	0.870
TP	0.998	0.989
$SD_{1}$	0.965	0.887
$SD_{2}$	0.998	0.993
$SD_{1}/SD_{2}$	0.767	0.732
EA	0.990	0.958
VAI	0.861	0.798
VLI	0.998	0.993

**Table 7:** Pearson’s linear correlation coefficient of HRV indices obtained on ECG and GCG signals.

HRV Index	ρ (Full Signals)	ρ (Signals Divided into 179 s Windows)
AVNN	1.000	1.000
SDNN	1.000	0.998
RMSSD	0.998	0.990
pNN50	0.997	0.993
VLF	1.000	0.998
LF	1.000	0.996
HF	0.998	0.995
LF/HF	0.987	0.937
TP	1.000	0.998
$SD_{1}$	0.998	0.990
$SD_{2}$	1.000	0.999
$SD_{1}/SD_{2}$	0.984	0.957
EA	0.999	0.995
VAI	0.995	0.991
VLI	1.000	0.999

## Abbreviations

The following abbreviations are used in this manuscript:

HRV	Heart rate variability
ECG	Electrocardiography, electrocardiogram
SCG	Seismocardiography
GCG	Gyrocardiography
MEMS	Microelectromechanical systems
MRI	Magnetic Resonance Imaging
EMD	Empirical Mode Decomposition
IMF	Intrinsic Mode Function
NN	The interval between consecutive normal heartbeats
FIR	Fininte impulse response [filter]
AO	Aortic valve opening [wave]
RSA	Respiratory sinus arrhythmia
SNR	Signal-to-noise [ratio]
AVNN	Mean inter-beat interval
SDNN	Standard deviation of all interbeat intervals
RMSSD	Root mean square of differences (RMSSD) of successive inter-beat intervals
pNN50	The proportion of the number of pairs of successive differences greater than 50 ms divided by total number of normal inter-beat intervals
VLF	The power of very low frequency band (0.0033–0.04 Hz) of HRV spectrum
LF	The power of low frequency band (0.04–0.15 Hz) of HRV spectrum
HF	The power of high frequency band (0.15–0.4 Hz) of HRV spectrum
LF/HF	LF/HF ratio
TP	Total power of HRV spectrum (up to 0.4 Hz)
$SD_{1}$	The width of the ellipse which containes the scatter points of Poincaré map
$SD_{2}$	The length of the ellipse which containes the scatter points of Poincaré map
$SD_{1}/SD_{2}$	$SD_{1}$ to $SD_{2}$ ratio
EA	The area of the ellipse which containes the scatter points of Poincaré map
VAI	Vector angular index
VLI	Vector length index
ρ	Pearson’s linear correlation coefficient
